# 

**Published:** 1910-07

**Authors:** 


					﻿PUBLISHED MONTHLY.	ATLANTA	SUBSCRIPTION $1.00
JOURNAL-RECe^xt?
OF MI DICirQl:1	'
»( . '
(Atlanta Medical and Surgical Journal, Established 1855. -)-E£f
and Southern Medical Record, Established 1870.	\3vlll ICdl
Vol. LVI.	Atlanta, Ga., July, 1910.	No. 4
				

